# Radiosensitization Effect of Nedaplatin on Nasopharyngeal Carcinoma Cells in Different Status of Epstein-Barr Virus Infection

**DOI:** 10.1155/2014/713674

**Published:** 2014-05-12

**Authors:** Li Yin, Jing Wu, Jianfeng Wu, Jinjun Ye, Xuesong Jiang, Meng Chen, Dejun Wang, Xue Wang, Dan Zong, Jiajia Gu, Junying Zhang, Jianzhong Wu, Lin Xu, Xia He, Wenjie Guo

**Affiliations:** ^1^Jiangsu Cancer Hospital, Nanjing Medical University, Nanjing 210009, China; ^2^Jiangsu Key Laboratory of Molecular and Translational Cancer Research, Nanjing 210009, China; ^3^Jilin Cancer Hospital, Changchun 130012, China; ^4^Department of Oncology, Xuzhou Medical College, Xuzhou 221004, China

## Abstract

This study aims to evaluate the radiosensitization effect of nedaplatin on nasopharyngeal carcinoma (NPC) cell lines with different Epstein-Barr virus (EBV) status. Human NPC cell lines CNE-2 (EBV-negative) and C666 (EBV-positive) were treated with 0–100 **μ**g/mL nedaplatin, and inhibitory effects on cell viability and IC_50_ were calculated by MTS assay. We assessed changes in radiosensitivity of cells by MTS and colony formation assays, and detected the apoptosis index and changes in cell cycle by flow cytometry. MTS assay showed that nedaplatin caused significant cytotoxicity in CNE-2 and C666 cells in a time- and dose-dependent manner. After 24 h, nedaplatin inhibited growth of CNE-2 and C666 cells with IC_50_ values of 34.32 and 63.69 **μ**g/mL, respectively. Compared with radiation alone, nedaplatin enhanced the radiation effect on both cell lines. Nedaplatin markedly increased apoptosis and cell cycle arrest in G2/M phase. Nedaplatin radiosensitized human NPC cells CNE-2 and C666, with a significantly greater effect on the former. The mechanisms of radiosensitization include induction of apoptosis and enhancement of cell cycle arrest in G2/M phase.

## 1. Introduction

Nasopharyngeal carcinoma (NPC) is an epithelial malignancy that is common in regions of Southeast Asia and Southern China [1]. As symptoms are insidious in the early stage, the majority of patients are diagnosed at an advanced stage. At present, concurrent chemoradiotherapy is the standard treatment for locally advanced NPC, and platinum-based chemotherapy is used most commonly [2]. Epstein-Barr virus (EBV) infection is a risk factor for NPC. Some studies have shown that EBV infection plays an important role in oncogenesis and development of NPC and that it also correlates with tumor load [3, 4].

Nedaplatin is a second-generation platinum drug, which has a radiosensitization effect on NPC [5]. However, the mechanisms of radiosensitization and whether it differs according to EBV infection status have not been reported. This is believed to be the first study describing the different radiosensitization effect at different stages of EBV infection and the mechanism involved.

## 2. Materials and Methods

### 2.1. Cell Culture and Drugs

Nedaplatin (Jiangsu Aosaikang Pharmaceutical Co. Ltd., China) was dissolved in 0.9% NaCl solution. Human NPC cells (Cancer Institute of Sun Yat-sen University, China) CNE-2 (EBV-negative) and C666 (EBV-positive) were maintained in RPMI-1640 medium (10% fetal bovine serum, 100 U/mL penicillin, and 0.1 mg/mL streptomycin). These cells were incubated at 37°C in a humidified atmosphere of 5% CO_2_.

### 2.2. MTS Cell Viability Assay

Cells were plated at a density of 2 × 10^3^ cells/mL in 96-well plates and allowed to attach for 24 h, resulting in log phase growth at the time of drug treatment. Nedaplatin (0, 0.25, 0.5, 1, 2, 5, 10, 20, 50, and 100 *μ*g/mL) was added to the wells, and cell viability was measured after 24, 48, and 72 h using an MTS assay (Promega, Madison, WI, USA) with a microplate reader at 490 nm [6]. Cell viability was expressed as a percentage of the value for control cultures. The cytotoxic effects of NDP on cells were expressed as 50% inhibitory concentration (IC_50_) values, which were calculated by SPSS software. All experiments were carried out in triplicate.

### 2.3. Colony Formation Assay

Cells (from 100 to 1 × 10^5^ cells) in 60 × 15 mm culture dish were seeded in triplicate and divided into six groups: control group (C group), radiotherapy group (RT group), chemotherapy group 1 (NDP1 group), chemotherapy group 2 (NDP2 group), combination group 1 (RT + NDP1 group), and combination group 2 (RT + NDP2 group). Concentrations of nedaplatin in the NDP1 and RT + NDP1 groups (IC_5_) were 0.34 *μ*g/mL (CNE-2) and 0.69 *μ*g/mL (C666) and in the NDP2 and RT + NDP2 groups (IC_10_) 0.64 *μ*g/mL (CNE-2) and 1.27 *μ*g/mL (C666), respectively. After allowing cells to attach to the dishes, cells of each group were treated with nedaplatin solution of corresponding concentration, whereas cells in the C and RT groups were without nedaplatin solution. After culture for a further 24 h and being replaced with serum containing RPMI-1640 medium, cells were radiated at 0, 2, 4, 6, and 8 Gy. Cells were incubated for 14 d to allow colony growth, and colonies were stained with Giemsa stain. Colonies containing ≥50 cells were counted and the plating efficiency was calculated by dividing the average number of colonies per dish by the number of cells plated. Survival fraction (SF) was calculated by normalization to the plating efficiency of appropriate control groups [7]. The above procedures were repeated three times.

### 2.4. Flow Cytometry

Cells were digested by 0.25% trypsin, seeded in six-well plates (5 × 10^5^ cells/well), and placed in nedaplatin solution at various concentrations (NDP1 and NDP2, see Section 2.3 above). For cell cycle analysis, cell culture was terminated after 24 h (Control, NDP1, and NDP2). For apoptosis analysis, cells were treated as described above (Control, RT, NDP1, NDP1 + RT, NDP2, and NDP2 + RT, see Section 2.3 above). Cells were collected by centrifugation at 1000 r/min, fixed with 70% ice-cold ethanol, adjusted to 1 × 10^9^/L, and stained with AnnexinV-fluorescein isothiocyanate (FITC)/propidium iodide (PI) for detecting apoptosis and PI for detecting cell cycle distribution (30 min, opaque background). Apoptosis and cell cycle distribution were detected by flow cytometry (Coulter EPICS XL, Coulter corp., USA) and data were analyzed by Multicycle software. The above procedures were also repeated three times.

### 2.5. Statistical Analysis

Statistical analysis was performed using GraphPad 5.0 and SPSS 18.0 software. All data were expressed as mean ± SD. Intergroup comparison was performed by one-way analysis of variance and pairwise comparison by least-significant difference and Student-Newman-Keuls (S-N-K) test. *P* < 0.05 was considered statistically significant.

## 3. Results

### 3.1. Cell Viability Explored by MTS Assay

**Figure 1:**
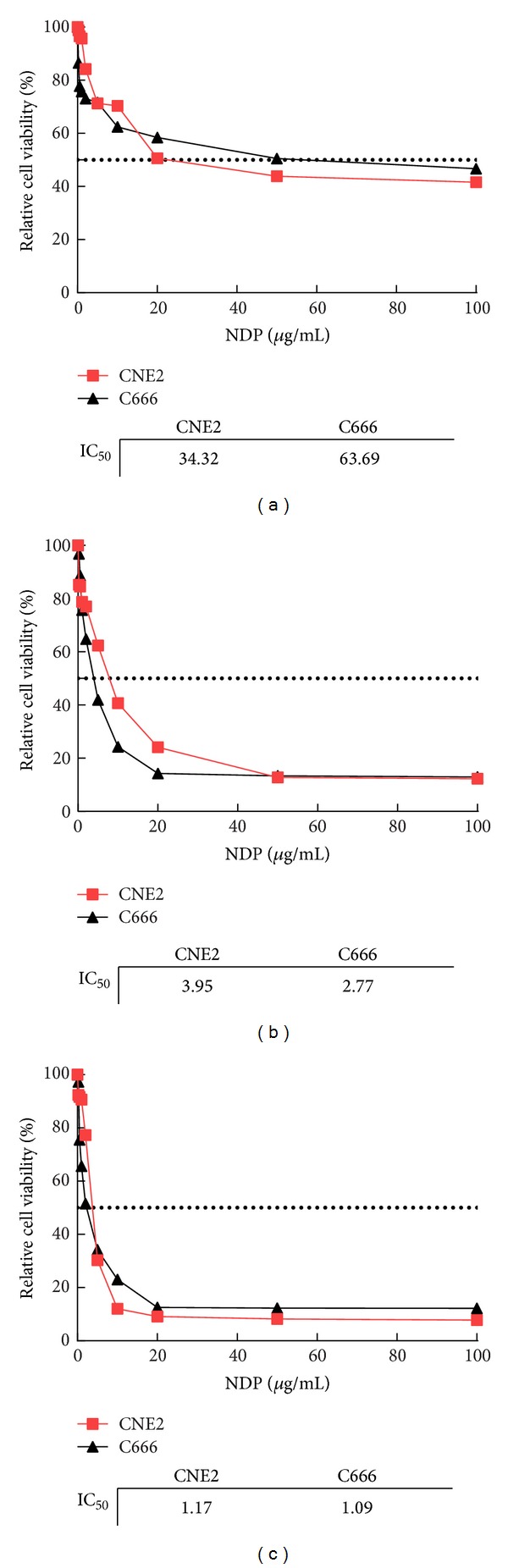
Toxicity of nedaplatin (NDP) on human NPC cells. NPC cells were incubated with various concentrations (0, 0.25, 0.5, 1, 2, 5, 10, 20, 50, and 100 *μ*g/mL) of NDP; cell viability was measured after 24 h (a), 48 h (b), or 72 h (c) by MTS assay.

In order to select the experimental concentrations for radiosensitivity research, the cytotoxicity of nedaplatin to CNE-2 and C666 cells was determined. Nedaplatin inhibited CNE-2 and C666 cell growth in a time- and dose-dependent manner (Figures 1(a)–1(c)). IC_50_ values for CNE-2 and C666 cell lines were 34.32 and 63.69 *μ*g/mL at 24 h, 3.95 and 2.77 *μ*g/mL at 48 h, and 1.17 and 1.09 *μ*g/mL at 72 h, respectively. At 24 h, IC_5_ values of CNE-2 and C666 were 0.34 and 0.69 *μ*g/mL, respectively, whereas IC_10_ values of CNE-2 and C666 were 0.64 and 1.27 *μ*g/mL.

### 3.2. Nedaplatin Enhances Antitumor Growth Effect of Radiation on CNE-2 and C666 Cells

**Figure 2:**
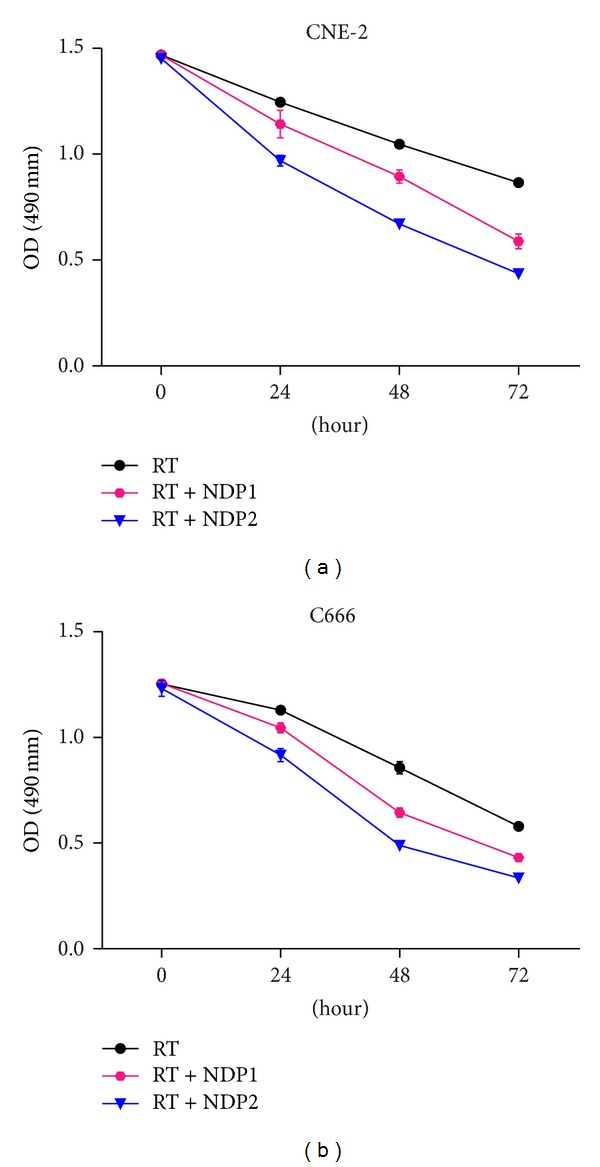
Effects of radiation (4 Gy), RT + NDP1 and RT + NDP2, on the proliferation of CNE2 (a) and C666 (b) cell lines in 72 hours. The growth inhibition curves of cells were generated by MTS assays from three replicate experiments (*P* < 0.05).

To evaluate whether nedaplatin pretreatment enhanced radiation-induced cell death, cell death by irradiation alone (RT) and in combination with nedaplatin (RT + NDP1 and RT + NDP2) was tested by MTS assay. As shown in Figure 2, the OD values of the RT, RT + NDP1, and RT + NDP2 groups in both cell lines decreased in a dose- and time-dependent manner after 4 Gy radiation. Similarly, the survival rates of the two cell lines in each group were shown in Table 1. Compared with the control group (radiation alone), cell survival rate decreased at 24 hours, 48 hours, and 72 hours in nedaplatin groups (one-way ANOVA, *P* < 0.05). Difference also existed between the two nedaplatin treatment groups (S-N-K test, *P* < 0.05).

### 3.3. Colony Formation Assay

**Figure 3:**
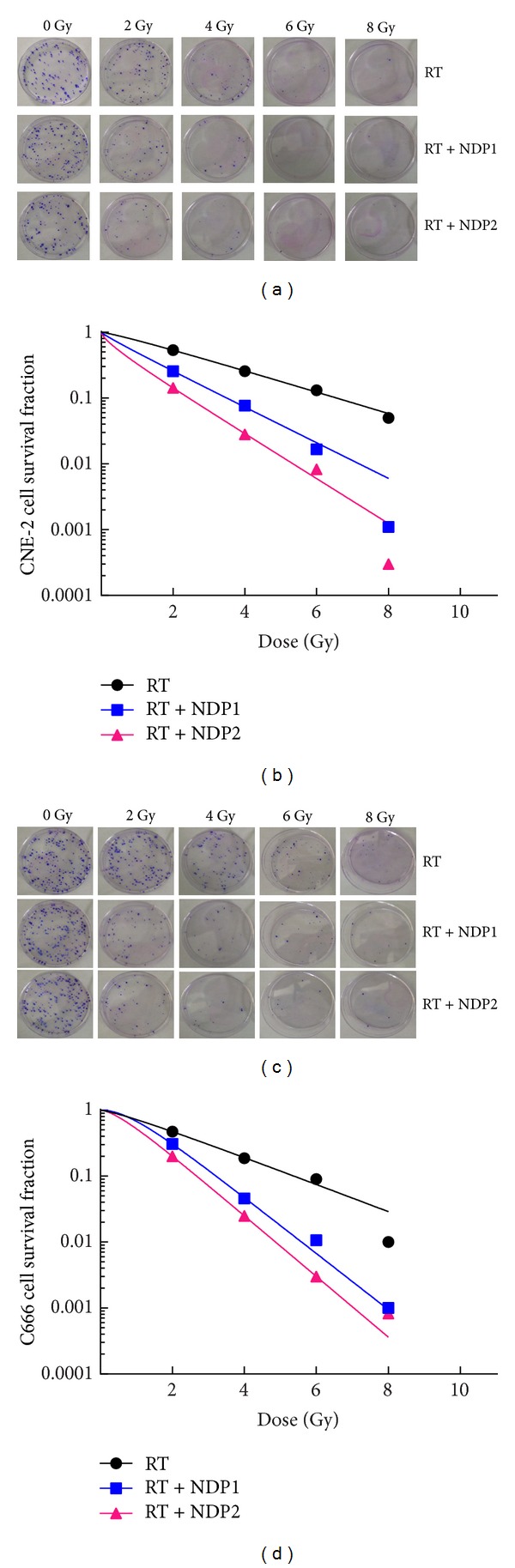
Survival curves of CNE-2 and C666 cells were generated according to SF by single-hit multitarget models. *R* ^2^ values were all above 0.998, which indicate the excellent performances in goodness of fit.

Cells were treated with nedaplatin for 24 h, and SF was calculated from the number of clones after radiation of 0, 2, 4, 6, and 8 Gy (Figures 3(a) and 3(c)). Compared with the RT group, the SF of the RT + NDP1 and RT + NDP2 groups decreased significantly with each dose of radiation. The survival curves of CNE-2 and C666 cells, which were generated according to SF by single-hit multitarget models (*R* ^2^ > 0.998), changed markedly (Figures 3(b) and 3(d), and Table 2). This indicated that the radiosensitivity of CNE-2 and C666 cells was significantly increased. The SF at 2 Gy (SF2) and the values of *D*_0_ and *D*_*q*_ in CNE-2 cells were 0.47, 2.20 Gy, and 0.55 Gy in the control group, respectively; 0.26, 1.45 Gy, and 0.03 Gy in the RT + NDP1 group (lower concentration); and 0.14, 1.01 Gy, and 0.01 Gy in the RT + NDP2 group (higher concentration). The corresponding values in C666 cells were 0.53, 2.55 Gy, and 0.84 Gy in the control group; 0.33, 1.75 Gy, and 0.43 Gy in the RT + NDP1 group; and 0.20, 1.21 Gy, and 0.17 Gy in the RT + NDP2 group (Table 2). Sensitization enhancement ratio (SER_*D*_0__) of CNE-2 and C666 cells in the RT + NDP1 group was 1.52 and 1.46, respectively, whereas in the RT + NDP2 group, it was 2.18 and 2.11.

### 3.4. Influence of Nedaplatin on Cell Cycle Distribution

Cell cycle analysis by flow cytometry showed that the proportion of cells in G2/M phase was significantly higher after nedaplatin treatment (one-way ANOVA, *P* < 0.05). There was also a significant difference between the two nedaplatin treatment groups (S-N-K test, *P* < 0.05). This indicated that nedaplatin influenced the distribution of NPC cells in each phase of the cell cycle (Table 3), especially at higher concentrations.

### 3.5. Apoptosis Induced by Nedaplatin at Different Concentrations

After 4 Gy radiation, the apoptosis rate of CNE-2 cells and C666 cells in the control, RT, NDP1, NDP2, RT + NDP1, and RT + NDP2 groups was showed, respectively, in Table 4. The percentage of apoptotic CNE-2 and C666 cells markedly increased with combined NDP and radiation (one-way ANOVA, *P* < 0.05). Apoptosis percentage was also higher in RT + NDP2 group than in RT + NDP1 group (S-N-K test, *P* < 0.05).

## 4. Discussion

Nedaplatin is one of the second-generation platinum drugs, which has similar molecular structure and pharmacological mechanisms to cisplatin. However, nedaplatin has lower hepatic and renal toxicity and has no cross-resistance with cisplatin [5]. In this study, we found that nedaplatin had excellent antineoplastic activity and exerted an inhibitory effect on NPC cells at different stages of EBV infection, in a time- and dose-dependent manner. Some studies [8–10] have demonstrated that concurrent chemoradiotherapy based on nedaplatin is safe, effective, and well tolerated by patients with carcinoma of the esophagus, lung, or uterine cervix and that nedaplatin has good radiosensitization effects. The present study is believed to be the first to compare the radiosensitization effect of nedaplatin on NPC cells at different stages of EBV infection. The effect of nedaplatin at low toxicity concentrations was detected by MTS and clone formation assays. We found that, at a given dose of radiation, the survival rate of cells in the combined chemoradiotherapy group was reduced significantly more than in the radiation alone group, and the reduction was correlated positively with the dose of nedaplatin. Thus, nedaplatin induces radiosensitization and can decrease the survival rate of carcinoma cells when administered concurrently with radiotherapy.

Clone formation assay is one of the most reliable methods to detect cell survival and is the gold standard for detecting radiosensitivity [11]. In our study, clone formation assay confirmed the results of the MTS assay. All radiosensitization parameters calculated by single-hit multitarget model decreased in the chemoradiotherapy group. The parameters included *D*_0_, *D*_*q*_, and *N*. *D*_0_ is the dose at which 63% of the cells are killed or 37% of the cells survive, and it represents the mean lethal dose or the mean inactivation dose. *N* is the number of radiosensitive areas in the cells. *D*_*q*_ is the width of the survival curve shoulder, in which cells can repair nonlethal damage. *D*_*q*_ represents the necessary dose in which cells are killed exponentially. In this study, we found that *D*_*q*_ and *N* decreased as the concentration of nedaplatin increased, which reduced repair of sublethal damage. This may be the radiosensitization mechanism of nedaplatin.

It has been demonstrated that oncogenesis of NPC correlates with EBV infection. Patients with NPC commonly have EBV infection. And EBV viral load is closely related to recurrence, metastasis, and therapeutic efficacy of NPC [12, 13]. Lo et al. [14] reported that the median EBV-DNA level in serum was significantly higher in advanced-stage compared with early-stage NPC patients and that after radiotherapy the median EBV-DNA level in patients who exhibited evidence of disease persistence or had developed distant metastases was also significantly higher than in patients with complete tumor regression. Some other studies showed similar results [15–17] and reported that, in NPC patients, high levels of serum EBV antibodies or EBV-DNA were negatively correlated with prognosis and overall survival.

In the present study, SF2 of C666 EBV-positive cells was higher than that of CNE-2 EBV-negative cells. This indicates that the former are more tolerant to radiation than the latter. SER_*D*_0__ of nedaplatin in CNE-2 cells was 1.52 (NDP1) and 2.18 (NDP2) whereas that in C666 cells was 1.46 (NDP1) and 2.11 (NDP2). This indicates a difference of radiosensitization effect of nedaplatin on NPC cells at different stages of EBV infection. The radiosensitization effect on CNE-2 cells was more effective than that on C666 cells. So, when nedaplatin is used for radiosensitization clinically, the status of EBV infection should be considered, and the dose of nedaplatin and radiation should be adjusted according to the EBV viral load in patients.

In previous studies, it has been demonstrated that tumor radiosensitivity is related to many factors, including tumor microenvironment, such as hypoxia, apoptosis, cell cycle regulation, and DNA repair dysfunction [18]. Cell cycle regulation is one of the most important determinants for tumor radiosensitivity. Cells in the G2/M phase are most sensitive to radiation whereas those in the S phase are resistant [19]. Researchers have achieved radiosensitization via cell cycle arrest in G2/M phase by taxanes or gene therapy [20, 21]. Apoptosis is the main mechanism of cell death after radiation, and the apoptosis index correlates positively with tumor radiosensitivity [22]. So enhancement of apoptosis is another mechanism in radiosensitization effects. In the present study, low concentrations of nedaplatin were chosen to reduce its cytotoxicity on radiosensitization results. The enhancement of apoptosis and the number of cells at G2/M phase due to nedaplatin may be the mechanism of radiosensitization. We also found enhancement of apoptosis index after combining nedaplatin and radiation and considered this to be another mechanism of radiosensitization. However, the correlation between radiosensitivity and the tendency towards cell cycle changes, and the interaction between cell cycle and apoptosis is complicated. Further research is needed to clarify these.

In conclusion, nedaplatin has a radiosensitization effect on human NPC cells CNE-2 and C666. The effect on CNE-2 cells is significantly more effective than that on C666 cells, which shows a dose-effect relationship. The mechanisms may include cell cycle arrest in G2/M phase, induction of apoptosis, and reduction of repair of sublethal damage.

## Figures and Tables

**Table 1:** Survival rates in each group after radiation of 4 Gy.

	CNE-2			C666		
	RT	RT + NDP1	RT + NDP2	RT	RT + NDP1	RT + NDP2
Survival rate (%)						
24 hrs	90.0 ± 0.3	80.2 ± 0.5	53.3 ± 0.7*	92.3 ± 0.2	77.7 ± 0.2*	53.8 ± 0.2*
48 hrs	76.7 ± 0.1	60.0 ± 0.6*	40.0 ± 0.2*	61.5 ± 0.7	44.6 ± 0.9*	34.6 ± 0.3*
72 hrs	66.7 ± 0.7	40.2 ± 0.4*	30.0 ± 0.6*	50.3 ± 0.3	36.2 ± 0.4*	23.1 ± 0.4*

*P < 0.05 was considered to indicate a statistically significant difference.

**Table 2:** Parameters of NPC cells in single-hit multitarget model under different conditions.

Parameters	CNE-2			C666		
	RT	RT + NDP1	RT + NDP2	RT	RT + NDP1	RT + NDP2
*D*_0_	2.20	1.45	1.01	2.55	1.75	1.21
*D*_*q*_	0.55	0.03	0.01	0.84	0.43	0.17
SF_2_	0.47	0.26	0.14	0.53	0.33	0.20
SER	—	1.52	2.18	—	1.46	2.11

*D*_0_: lethal radiation dose; *D*_*q*_: quasi-threshold dose; SF_2_: fraction of cells surviving after 2 Gy radiation; SER: radiosensitization ratio (sensitization enhancement ratio), calculated as *D*_0_ for the control group divided by *D*_0_ for the treatment group.

**Table 3:** Cell cycle distribution in each group with different NDP concentrations.

Proportion (%)	CNE-2			C666		
	Control	NDP1	NDP2	Control	NDP1	NDP2
G0/G1	47.77 ± 1.41	31.08 ± 1.21	25.03 ± 1.35	46.11 ± 2.70	37.48 ± 1.12	32.04 ± 1.20
S	48.74 ± 1.61	51.37 ± 0.54	42.88 ± 0.62	50.24 ± 2.17	48.77 ± 1.50	37.85 ± 1.44
G2/M	3.49 ± 0.25	17.55 ± 0.55*	32.09 ± 0.48*	3.65 ± 0.18	13.75 ± 0.51*	30.11 ± 0.77*

*indicates *P* < 0.05 in comparison between control and NDP groups in the two human NPC cell lines and between NDP1 and NDP2 groups.

**Table 4:** Apoptosis rate of two cell lines after radiation of 4 Gy.

	Control	RT	NDP1	NDP2	RT + NDP1	RT + NDP2
Apoptosis rate (%)						
CNE-2	2.30 ± 0.10	4.55 ± 0.12	2.50 ± 0.31	2.70 ± 0.18	9.02 ± 0.75*	14.55 ± 0.45*
C666	2.12 ± 0.23	4.02 ± 0.33	2.68 ± 0.40	2.77 ± 0.22	7.11 ± 0.32*	10.36 ± 0.58*

*indicates *P* < 0.05 in comparison between RT and RT + NDP groups in the two human NPC cell lines and between RT + NDP1 and RT + NDP2 groups.
